# Influence of Incorporating 5% Weight Titanium Oxide Nanoparticles on Flexural Strength, Micro-Hardness, Surface Roughness and Water Sorption of Dental Self-Cured Acrylic Resin

**DOI:** 10.3390/polym14183767

**Published:** 2022-09-08

**Authors:** Rasha M. Abdelraouf, Rania E. Bayoumi, Tamer M. Hamdy

**Affiliations:** 1 Biomaterials Department, Faculty of Dentistry, Cairo University, Cairo 11553, Egypt; 2 Biomaterials Department, Faculty of Dentistry (Girls), Azhar University, Cairo 11754, Egypt; 3 Restorative and Dental Materials Department, Oral and Dental Research Institute, National Research Centre (NRC), El Bohouth St., Dokki, Giza 12622, Egypt

**Keywords:** polymethyl methacrylate (PMMA), flexural strength, micro-hardness, surface roughness, water sorption, titanium oxide nanoparticles, self-cure acrylic resin

## Abstract

Background: Polymethyl methacrylate (PMMA) is used in fabricating acrylic denture bases. Repairing a fractured acrylic denture base can be done by self-cured PMMA, yet this is still a weak point after repair. The aim of this study was to evaluate the effect of incorporating 5% weight titanium oxide nanoparticles (TiO_2_) to self-cured PMMA on flexural strength, surface micro-hardness, roughness, and water sorption. Methods: A total of 160 acrylic–resin specimens were used in this study. They were divided in two main groups; (a) control group, prepared by mixing self-cured PMMA powder to its liquid monomer, (b) treated group, prepared by blending 5% weight TiO_2_ nanoparticles to self-cured PMMA powder then this blend was mixed with the liquid monomer. Flexure strength, surface micro-hardness, roughness, and water sorption were evaluated. Data were analyzed using independent sample *t*-tests (*p* ≤ 0.05). Results: There was a significant increase in the flexural strength of PMMA of the treated group after the addition of TiO_2_ (137.6 MPa) compared with the control (75.4 MPa) (*p* ≤ 0.001). No significant difference between the two groups in terms of micro-hardness (*p* = 0.385) and surface roughness (*p* = 0.269). Water sorption showed a significant reduction in the treated group (*p* ≤ 0.001). Conclusions: Addition of 5% weight TiO_2_ nanoparticles to the self-cured acrylic resin improved its flexural strength and reduced its water-sorption without impairing the surface micro-hardness and roughness.

## 1. Introduction

Polymers represent a widespread material used in the dental field [1,2,3]. Acrylic resins are one of the most used materials for removable denture base constructions. Heat or self-cured acrylic resins are composed of polymethyl methacrylate (PMMA). Denture base fabrication depends mainly on heat-cured acrylic resin. Denture repairs are carried out by self-cured acrylic resin to avoid warpage and destruction of the fractured parts of the denture [4,5]. Fractures of denture bases may happen inside the mouth mostly due to high flexural stresses occurring during mastication on a long-term service [6]. However, fracture outside the mouth is mainly by accident such as the dropping of the denture [7]. Repairing or rebasing of the fractured denture base by self-cured (chemical cured) acrylic resins is considered a good approach, thus preserving time and costs if deciding to construct a new denture [8].

Acrylic denture base fractures mainly arise from impact and or flexure forces. An impact force may cause denture fracture when sudden dropping occurs. Fracture of dentures due to flexure force could happen on repeated flexing [9]. Despite the desired properties of PMMA, there is a great demand for improvement of their mechanical properties [10].The minimal standard limit for flexural strength of the acrylic resin should be no less than 50 MPa according to the ISO international standard for dentistry base polymers [11]. The higher the flexural strength, the more prolonged the clinical service lifespan of the denture [12].

Self-cured acrylic resin is one of the most used denture repair materials. The flexural strength of self-cured acrylic resins is lower than that of the heat-cured type; therefore, recurring fractures can be predicted. Concern should be shown to modifying the repaired dentures through reinforcement of the repairing materials [13].

There have been many attempts to reinforce PMMA-based materials by incorporating fillers into their composition. The use of fibers, ceramics or metals to PMMA produces beneficial effects [14,15,16,17,18]. The introduction of nanotechnology offers a new approach for the enhancement of the mechanical properties of polymers [10,19,20]. Particularly, the incorporation of hard and strong nano-filler particles into the polymeric matrix affects significantly the mechanical properties and structural integrity of the polymeric-based dental materials [14,15].

Heat-cured PMMA acrylic resins tried to be reinforced in previous studies. However, there are few studies conducted about the reinforcement of self-cured PMMA acrylic [21,22,23,24]. Modifications have been done to improve the flexural strength of self-cured acrylic denture base material to counteract the problem of their great possibility to fracture [25]. One approach to strengthening the PMMA material is by addition of rubber in what is called a high-impact denture base to avoid fracture under impact force [26]. Another approach depends on the addition of reinforcement materials such as aluminum oxides and glass fiber nanoparticles [25,27,28].

Although there are many types of nano-metals such as silver and cobalt–chromium, titanium dioxide (TiO_2_) nanoparticles have a great value due to their cardinal features, such as: being non-toxic; safe; chemically inert; having antimicrobial properties; having high flexural strength; fracture toughness; and micro-hardness [29,30,31]. 

Flexural strength of acrylic denture bases is considered as an important factor of clinical failure [30,32]. Therefore, a high mechanical strength property of denture base materials is considered the most important requirement for successful denture base materials [33]. Micro-hardness indicates the abrasiveness of the denture base material. The surface properties of acrylic denture bases are affected by micro-hardness, which denote resistance to scratching during service and upon denture cleansing [34]. Moreover, surface roughness and irregularities of a PMMA denture base promotes stains and plaque accumulation affecting negatively the aesthetics and biological properties of acrylic denture with time [35]. In addition, water sorption by polymeric matrix of denture has a detrimental effect on mechanical properties of acrylic denture base, thus it should be minimal [18].

There is insufficient data on the impact of the addition of TiO_2_ nanoparticle on the mechanical and physical properties of the self-cured acrylic resin used for denture repair or rebase. Thus, the aim of this study was to evaluate the effect of incorporation of 5% weight TiO_2_ nanoparticles on the mechanical properties of self-cured PMMA regarding flexural strength, surface micro-hardness, roughness, and water sorption.

The null hypothesis is that addition of 5% TiO_2_ nanoparticles does not change the flexural strength of the self-cured PMMA in comparison to the unmodified group.

## 2. Materials and Methods

Self-cured acrylic resin was used in this study (Acrostone Cold Cure Acrylic Resin, Acrostone Co., London, UK). TiO_2_ nanoparticles were purchased from Sigma Aldrich (Sigma-Aldrich Chemie GmbH, Schnelldorf, Germany) with an average particle size < 25 nm. A total of 160 acrylic–resin specimens were used in this study. They were divided in two main groups; (a) the control group was prepared by mixing self-cured PMMA powder to its liquid monomer in ratio 3:1 by volume according to the manufacturer’s instructions, (b) the treated group was prepared by adding 5% TiO_2_ nanoparticles to self-cured PMMA powder (95%) (by weight). Manual mixing was performed by blending the two powders by rotation motion for 10 min using a spatula, followed by shaking in a sealed container for an additional 10 min. Then, the blended powder was added as one unit to liquid monomer in a ratio 3:1 by volume as previously mentioned.

When the mixed acrylic resin reached the dough stage, it was packed in special molds according to the test. According to the manufacture, the polymerization process was carried out for 10 min, after that the prepared specimens were removed out of the molds and visually inspected to verify the presence of a smooth and flat surface, showing no defects, voids, or porosity; otherwise, they were discarded. After that, the specimens were immersed in distilled water at 37 °C for 24 h before testing [18,36].

The parameters measured in this study were flexural strength, surface micro-hardness, roughness and water sorption. For each test, 20 specimens were prepared from each group (control and treated).

### 2.1. Flexural Strength Test

The flexural strength was determined by a 3-point bending flexural strength test in accordance with ISO 20795-1 [11,18]. Specimens of 64 mm (length) × 10 mm (width) × 3.3 mm (thickness) were prepared using stainless [11,18]. The specimens were investigated using a universal testing machine (Model 3345; Instron Industrial Products, Norwood, MA, USA) with two supports 20 mm apart, at a crosshead speed of 5 mm/min. The load at fracture was recorded in Newtons (N) and the flexural strength (FS) was calculated in MPa with the following equation [18,37]:

FS = PL/wb^2^, (1)

where; (P) is the maximum load at fracture, (L) is the distance between the supports (=20 mm), (w) is the specimen thickness, and (b) is the height. The specimens’ measurement was done with a digital caliper.

### 2.2. Micro-Hardness Test

Surface micro-hardness was determined using Digital Vickers hardness tester (NEXUS 400TM, INNOVATEST, model no. 4503, Maastricht, The Netherlands). Specimens of dimension (65 mm × 10 mm × 2.5 mm) were fabricated in a stainless-steel mold [27,38]. The indentations were made within 20 s from the loading 500 g at 20× magnification [27,38].

The Vickers micro-hardness number (VHN) value was calculated automatically using the equation:

VHN = 1.8544 P/d^2^ (2)

where; (P) is the applied force in kilogram, (d) is the mean of the two diagonals gained from the indentation in mm.

### 2.3. Surface Examination and Surface Roughness Test

The morphology of the surfaces was examined using non-destructive environmental scanning electron microscopy (Model Quanta 250 FEG, FEI company, Eindhoven, The Netherlands) with 200× magnification [39,40]. Disc specimens were obtained from a stainless-steel mold (10 mm diameter × 2 mm height) [41]. The same specimens were then used for the surface roughness test using the SEM micrograph [42,43,44]. SEM images were converted into 3rd dimension images by imaging analysis system Scandium Solution Height (Olympus soft imaging solutions, GMBH, Muenster, Germany). The average surface roughness values of each specimen (Ra) were recorded.

### 2.4. Water Sorption Evaluation

The water sorption test was accomplished by the preparation of disc specimens (50 mm diameter and 0.5 mm in thickness) [31]. Specimens were placed inside a desiccator containing a dried silica gel and stored in an incubator (CBM, S.r.l. Medical Equipment, 2431/V, Cremona, Italy) at 37 ± 1° for 24 h. Specimens were weighed using an electronic balance (Adam equipment 4 digits precision weighing balance, Adam Equipment Inc., Oxford, UK) until their weight was constant. The initial weight was termed as W1 (μg). Specimens were then immersed in distilled water at 37 ± 1 °C and stored into the incubator for 15 days [45]. Specimens were removed from the water, gently dried with an absorbent paper and air sprayed for 15 s. Specimens were weighed again and were termed as W2 (μg).

The diameter and thickness of each specimen was measured by digital caliper. The volume (V) of each specimen was calculated following equations:

V = π × r^2^ × h (3)

where r is the mean specimen radius (diameter/2) in millimeters and h is the mean specimen thickness in millimeters. 

The water sorption was attained from the difference between the initial and wet weighing (W2 − W1). The values of water sorption (Wsp) in μg/mm^3^, for each specimen were calculated using the following equations:

Wsp = (W2 − W1)/V(4)

### 2.5. Statistical Analysis

The statistical analysis was performed using the Statistical Package for the Social Sciences (IBM SPSS Statistics for Windows, Version 23.0, IBM Corporation, Armonk, NY, USA). An independent sample t-test was used to compare mean values of flexural strength between two groups (control PMMA and treated PMMA with TiO_2_). The significance level was set at *p* ≤ 0.05. Similarly, the same test was used to compare the results of micro-hardness as well as surface roughness and water sorption between the two groups.

## 3. Results

**Figure 1:**
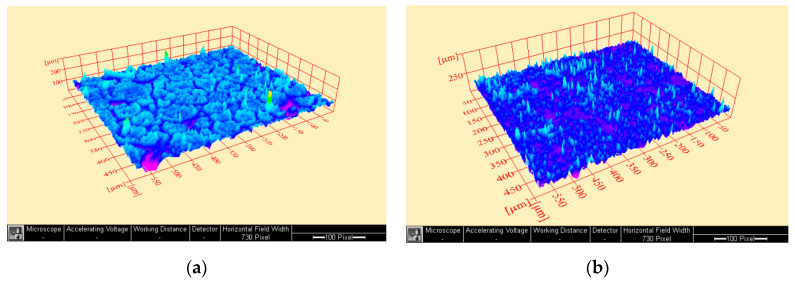
Representative 3-D surface roughness images (Ra) (**a**) control group; (**b**) Treated group.

**Figure 2:**
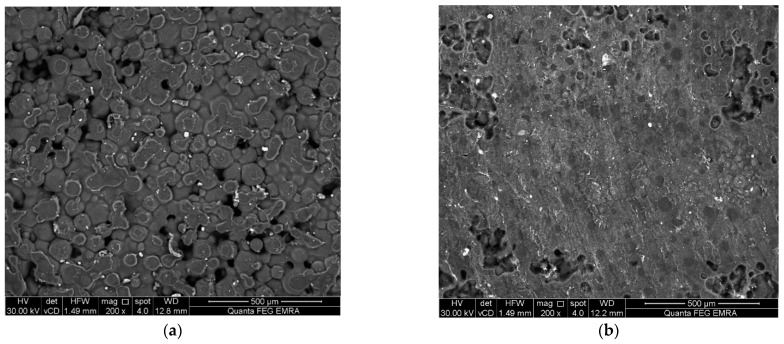
Representative SEM micrographs (200× magnification) (**a**) control group; (**b**) Treated group.

**Figure 3:**
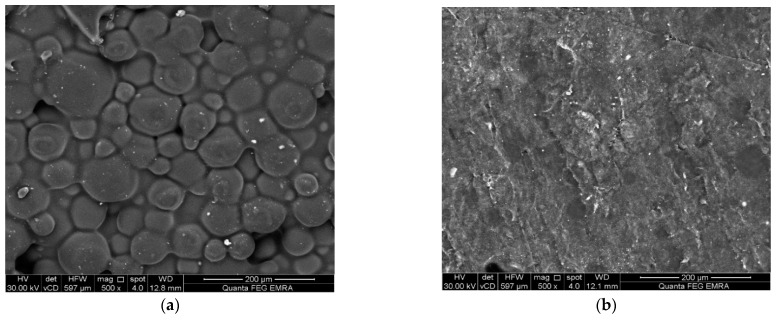
Representative SEM micrographs (500× magnification) (**a**) control group; (**b**) Treated group.

The mean, standard deviation values for flexural strength (MPa), Vickers micro-hardness number (VHN), SEM surface examination images, 3-D surface roughness (Ra), and water sorption (μg/mm^3^) are presented in Table 1 and Figure 1, Figure 2 and Figure 3. There was a significant increase in flexural strength of PMMA of the treated group after the addition of TiO_2_ (137.6 MPa) compared with the control group (75.4 MPa). No significant difference was seen between the two groups in results of micro-hardness as well as surface roughness. On the other hand, the water sorption showed a significant reduction after the addition of TiO_2_ (0.36 µg/mm^3^), in comparison to the control group.

Moreover, SEM micrograph showed that the surface of non-treated specimens (control) exhibited a honeycomb appearance and porous surface. Meanwhile, the treated specimens (filled with TiO_2_) demonstrated a less porous surface compared with the control.

## 4. Discussion

The effect of addition of TiO_2_ nanoparticle on flexural strength of PMMA depends on both concentration of nanoparticles and their interaction with the acrylic resin polymer. The ideal concentration of the fillers is still under investigation [46].

A previous study investigating the interaction between TiO_2_ nanoparticle and PMMA found that TiO_2_ nanoparticles interact chemically with PMMA through their reaction with the ester functional group COOR (oxygen double-bound to carbon–carbonyl along with an OR group attached to the same carbon). The ester is a carboxylic acid derivative in which the OH is replaced by and OR (oxygen reaction variable). Moreover, the loading of TiO_2_ nanoparticles within the resinous matrix hinders polymer chain movements due to the strong bond between the TiO_2_ nanoparticle and PMMA providing a cross-linking action [47].

In this study, the null hypothesis is rejected, as the treated PMMA by addition of 5% weight TiO_2_ nanoparticles showed higher flexural strength than the control group. These values exceed the minimum requirements of flexural strength which is not less than 50 MPa [11]. This may be due to the chemical and physical interaction between TiO_2_ nanoparticles and PMMA as mentioned earlier.

Surface hardness and roughness of the denture are two crucial factors to avoid scratching of denture on service or collection of food, bacterial and fungal to avoid their adhesion to denture surface with subsequent candidiasis and inflammation [48]. Recent investigation suggested the addition of 5% TiO_2_ nanoparticle which was considered suitable to raise conventional and high impact heat cured acrylic resin surface hardness to significant values [30,49].

The micro-hardness and surface roughness revealed no significant difference among all tested groups which may be attributed to the low concentration of the homogenously blended TiO_2_ nano-sized particles fillers within the polymer matrix. There was no statistically significant difference in the surface roughness values among all tested groups, but the reduced surface roughness in the treated groups may be beneficial to decrease the possibilities of stains and plaque accumulation.

The SEM micrograph of the control specimens displayed a honeycomb appearance with an irregular and porous structure. This may be attributed to the presence of pre-polymerized PMMA beads region as a major component of PMMA powder surrounded by in-situ PMMA which was formed by the polymerization reaction of methyl methacrylate monomer liquid. The SEM micrograph of treated specimens displayed a homogenous less porous structure. This can be explained by the uniform distribution of the titanium dioxide nanoparticle fillers within the polymer matrix.

The nature of the denture base resin material allows some degree of water sorption. Water molecules entrances and acts as a plasticizer that affects the dimensional stability and durability of the denture base [50]. The water sorption was evaluated by the increase in mass per unit volume. According to ISO standards 1567:1999 specification, water sorption for heat-cured or self-cured materials should not exceed 32 μg/mm^3^ [50,51].

The decrease in water sorption in the treated group may be attributed to the fact that the addition of TiO_2_ nanoparticles to acrylic–resin spaces reduces the ability of the treated PMMA denture base to absorb water molecules due to their cross-linking effects of TiO_2_ nanoparticles. Furthermore, TiO_2_ nanoparticles are considered an insoluble hydrophobic element [52].

Despite the rise in the flexural strength of PMMA modified by the addition of 5% TiO_2_ nanoparticles, the manual blending of the two powders may exert some limitations in the conducted research.

## 5. Conclusions

Addition of 5% weight TiO_2_ nanoparticles to dental self-cured acrylic PMMA resin improved its flexural strength and reduced its water sorption without the impairment of the surface micro-hardness and roughness.

## Figures and Tables

**Table 1:** Mean and standard deviation values of flexural strength, micro-hardness, surface rough-ness and water sorption tests of the two groups.

Test/Group	Control Group	Treated Group	*p* Value
Flexural strength (MPa)	75.4 ± 2.1	137.6 ± 3.2	≤0.001 *
Micro-hardness (VHN)	15 ± 1.3	15.7 ± 0.8	0.385
Surface roughness (Ra)	16 ± 2.8	13.7 ± 3.2	0.269
Water sorption (μg/mm^3^)	0.63 ± 0.01	0.36 ± 0.01	≤0.001 *

*: significant (*p* < 0.05).

## Data Availability

The data presented in this study are available on request from the corresponding author.
